# Foreign stingers: South American freshwater river stingrays *Potamotrygon* spp. established in Indonesia

**DOI:** 10.1038/s41598-023-34356-9

**Published:** 2023-05-04

**Authors:** Rikho Jerikho, Surya Gentha Akmal, Veryl Hasan, Yonvitner, Jindřich Novák, André Lincoln Barroso Magalhães, Alberto Maceda-Veiga, Michael F. Tlusty, Andrew L. Rhyne, Ondřej Slavík, Jiří Patoka

**Affiliations:** 1 Department of Zoology and Fisheries, Faculty of Agrobiology, Food and Natural Resources, Czech University of Life Science Prague, Kamýcká 129, 16500 Prague–Suchdol, Czech Republic; 2 Centre for Coastal and Marine Resources Studies, The Institute for Research and Community Service, IPB University, Bogor, 16680 Indonesia; 3 Department of Aquaculture, Faculty of Fisheries and Marine, Universitas Airlangga, Surabaya, 60115 Indonesia; 4 Programa de Pós-Graduação em Ecologia de Biomas Tropicais, Universidade Federal de Ouro Preto, Rua Diogo de Vasconcelos 122, Ouro Preto, Minas Gerais 35400-000 Brazil; 5 Departament de Biologia Evolutiva, Ecologia i Ciències Ambientals, Institut de Recerca de la Biodiversitat (IRBio), Universitat de Barcelona, 08028 Barcelona, Spain; 6 School for the Environment, University of Massachusetts Boston, Boston, MA 02125 USA; 7 Department of Biology, Marine Biology, and Environmental Science, Roger Williams University, One Old Ferry Road, Bristol, RI 02809 USA

**Keywords:** Biodiversity, Climate-change ecology, Environmental economics, Invasive species, Trauma, Ecological modelling

## Abstract

The pet trade is known to be one of the most important pathways of aquatic non-native species introduction and Indonesia is a significant trade partner. Popular ornamental South American river stingrays (*Potamotrygon* spp.) were introduced to Indonesia in the 1980s and the culture was established. Here we present a detailed Indonesian market and aquaculture survey, the volume of trade between January 2020 and June 2022, and the list of customer countries with the total amount of imported stingrays. Climate similarities between the native range of *P*. *motoro* and *P*. *jabuti* and Indonesia were analysed. A significant number of areas of Indonesian islands were identified as suitable for the establishment of this species. This was confirmed by the first record of probably established populations in the Brantas River (Java). In total 13 individuals including newborns were captured. The culture of potamotrygonid stingrays is unregulated in Indonesia, and the risk of the establishment of this predator and its potential spread is alarming for wildlife. Moreover, the first case of envenomation caused by *Potamotrygon* spp. in the wild outside of South America was recorded. The current condition is predicted as the ‘tip of the iceberg’ and continuous monitoring and mitigation of risks are strongly recommended.

## Introduction

Biological invasions cause extensive environmental and socio-economic losses worldwide^1–3^. The invasive species tend to have wide adaptability to different environmental conditions (e.g. wide temperature adaptability and oxygen requirement)^4^. Ornamental aquaculture and the related pet-trade industry are important sources of non-native species given the quantity and diversity of species cultured and transported across the globe^5–9^. The producers, traders, garden pond vendors and aquarium owners have been responsible for many introductions of ornamental creatures, some of which have established self-sustaining populations as invasive species^10–13^. Even if policymakers aim to mitigate biological invasion risks by regulating the pet trade, there are examples of failure^14,15^ including up to seven years after the species was banned for sale^16^.

Since most of the species exploited in ornamental aquaculture are thermophilous (i.e., warmth-loving species)^5^, the most vulnerable regions threatened by putative invasions are the tropics (documented by several examples of introduced ornamentals)^2,17–20^. These introductions may also lead to the “Biodiversity Conservation Paradox” which occurs when aquarium species that are endangered in their native range will behave as invaders when introduced elsewhere such as the giant arapaima or pirarucu (*Arapaima gigas*) in Indonesia^21^.

Another popular large aquarium fishes are the South American freshwater stingrays from the genus *Potamotrygon* (family Potamotrygonidae, subfamily Potamotrygoninae)^22–24^. Despite the high price, these stingrays are sold as ornamentals because they are attractive and easy to keep in captivity^25,26^. Ornamental fishing for more than four decades in Amazon catches these stingrays^27^. The genus *Potamotrygon* is the most diverse^28,29^. The popularity of *Potamotrygon* spp. also stems from being the only elasmobranchs completely adapted for living in a freshwater environment as well as their reproductive mode, described as matrotrophic viviparity^30,31^. The first record of an established introduced potamotrygonid population elsewhere out of the native range was in Singapore where the ocellate river stingray (*Potamotrygon motoro* (Müller & Henle, 1841)) was recorded in 2009^25^. From all potamotrygonids, *P*. *motoro* is the most widespread species occurring in most freshwaters in South America, including the Amazon, Mearim, Orinoco, Paraná-Paraguay, and Uruguay basins including most regions of Brazil^32^.

Potamotrygonins represent an important part of the Neotropical freshwater ichthyofauna and are collected in the wild and overharvested in some regions in South America such as Brazil, Peru, Colombia, Venezuela^24,33–35^. From about 42 species of the family Potamotrygonidae^35^, only seven species are newly fully protected by CITES Convention. Following amendments implementation of CITES CoP 19, *Potamotrygon albimaculata*, bigtooth river stingray *P*. *henlei*, *P*. *jabuti*, white-blotched river stingray *P*. *leopoldi*, *P*. *marquesi*, Parnaiba river stingray *P*. *signata* and *P*. *wallacei* were included in Appendix II of the Convention, valid from 23 February 2023 (NOTIFICATION TO THE PARTIES, Secretariat of the Convention on International Trade in Endangered Species of Wild Fauna and Flora (CITES), No. 2023/015, Geneva, 10 February 2023, cites.org). Moreover, followed populations are classified in Appendix III: The Colombian populations of discus ray *Paratrygon aiereba*, thorny river stingray *Potamotrygon constellata*, Magdalena river stingray *P*. *magdalenae*, ocellate river stingray *P*. *motoro*, smooth back river stingray *P. orbignyi*, rosette river stingray *P*. *schroederi*, raspy river stingray *P*. *scobina*, and Maracaibo river stingray *P*. *yepezi*, and the Brazilian populations of *Potamotrygon* spp. not included in Appendix II. Certain local populations of selected species from Colombia (*Paratrygon aiereba, Potamotrygon constellata*, *P*. *magdalenae*, *P*. *motoro*, *P*. *orbignyi*, *P*. *schroederi*, *P*. *scobina*, and *P*. *yepezi*), and Brazil (*Potamotrygon* spp.) are listed in CITES Appendix C in European Union (Commission Regulation (EU) No. 2021/2280 of 16 December 2021 amending Council Regulation (EC) No. 338/97 on the protection of species of wild fauna and flora by regulating trade therein, and Commission Regulation (EC) No. 865/2006 laying down detailed rules concerning the implementation of Council Regulation (EC) No. 338/97). The amendment of the European Annexes to Council Regulation (EC) 338/97 is currently in process. In total, many of formally described species of five genera of Neotropical freshwater stingrays still remain not protected by the Convention and their proposals to CITES were rejected due to insufficient trade and population status data^22^.

Unfortunately, the regulations and restrictions are ineffective in many cases (in part also for the aforementioned reasons) and these stingrays are traded illegally^36^. Since they are economically valuable fishes (more than 68,000 of wild-collected individuals were exported from Brazil in 2003–2016; WCS Position Statement CITES CoP17—Johannesburg, South Africa 2016), their commercial culture was established in many countries in Asia^25^. One of the leading producers and exporters of ornamental aquatic species is Indonesia^6,37,38^. Regarding the tropical climate in this region, numerous non-native ornamental aquarium species have established new populations there after accidental or deliberate introduction^11,17,19,21,39^. Despite the exported shipments, ornamental animals are also traded in huge quantities via domestic markets^19,40^. While regulations for the management of non-native fish species and their associated trade exist^41^, the current legislative framework is mostly ineffective in this regard^14^. Thus, there is a pressing need to predict the most vulnerable habitats to invasions by ornamental aquarium species.

The goal of this study is to inform the invasion potential of the very popular South American freshwater stingray *Potamotrygon* spp. in tropical regions such as Indonesia. Given that the spread of invasive species of ornamental origin in Indonesia is likely, we surveyed the trade evidence and screened the online market for the presence of stingrays for sale and natural waters through local fishermen for the location of potentially already established invasive populations to address the risks they pose for the native environment, biota, and humans. Since the climate is among the major factors explaining the successful establishment of invasive ornamental fish species^42,43^, we used species distribution models with climatic variables to predict the regions of Indonesia potentially vulnerable to this invasion.

## Results

### Domestic trade

**Figure 1:**
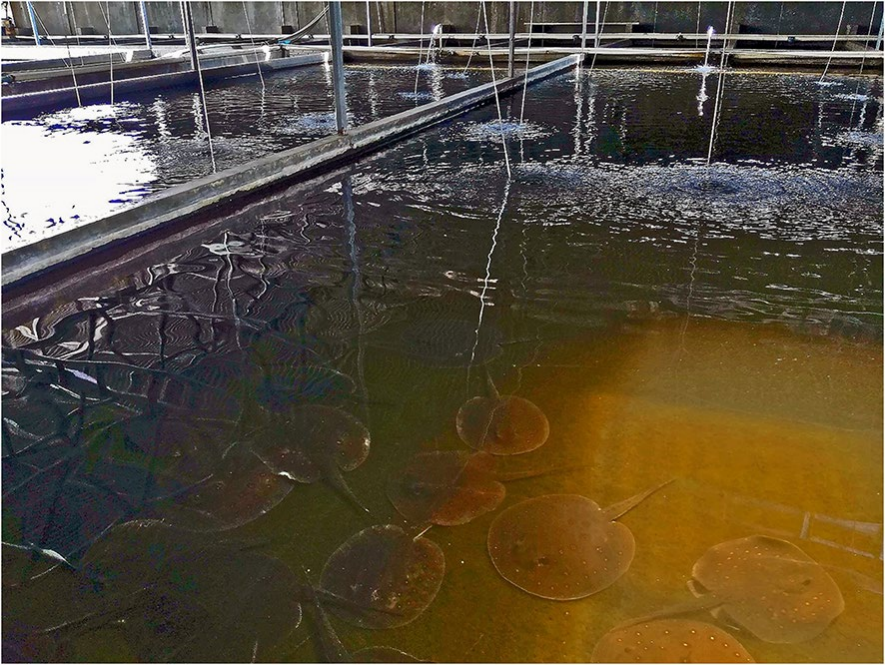
Local farm in Cirebon City (West Java) where ornamental fish including *Potamotrygon motoro* and *P*. *leopoldi* are produced in huge quantities.

In total, 66 online sale offers of *Potamotrygon* spp. (advertised as *P*. *motoro*) were recorded in Indonesia (Java, Sumatra and Bali) within January 2022. Fish were offered for 700,000–20,000,000 IDR (47.8–1365.9 USD) per individual in quantity 1–100 individuals. The stores are located on the islands of Java, Sumatra and Bali. The price depends on the colouration patterns and size of the offered individual (10–36 cm in disc diameter). The albino phenotype has never been recorded in Indonesia. The local name in Bahasa Indonesia is ‘Pari Motoro’. Moreover, there are also other species of the genus *Potamotrygon* offered via pet trade in this country such as the white-blotched river stingray (*P*. *leopoldi*). These stingrays are produced in local farms in Indonesia (Fig. 1).

### Climate matching

**Figure 2:**
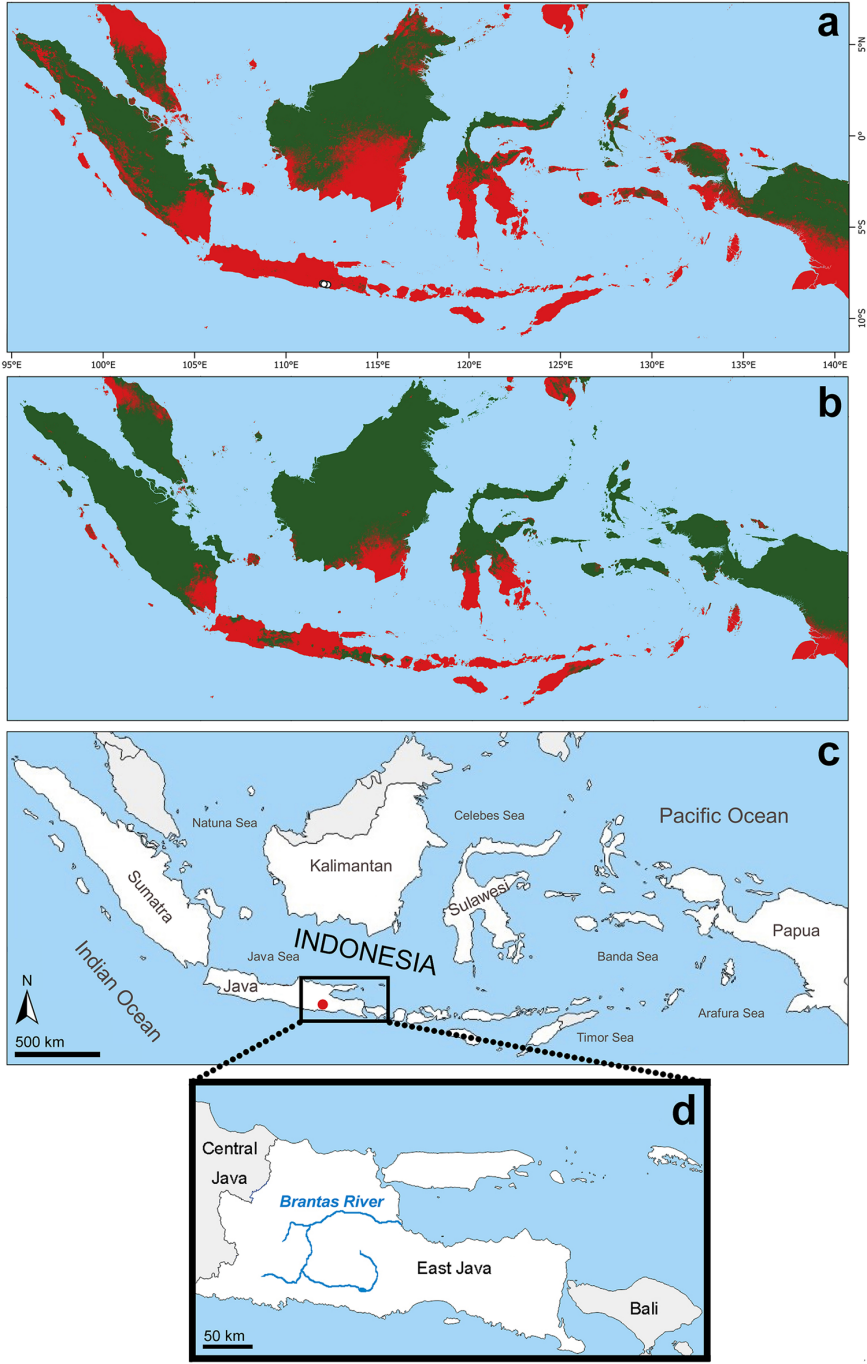
Map derived from the MaxEnt model (v. 3.4.4, https://biodiversityinformatics.amnh.org/open_source/maxent/) for the possible establishment of *Potamotrygon motoro* (**a**) and *P*. *jabuti* (**b**) in Indonesia. The red colour indicates areas suitable for establishment due to climatic similarity with the native range. The white dots show locations used for model training (**a**); the red dot indicates the Brantas River where the first potentially established population in Indonesia was found (**c**); detail of East Java Province with the Brantas River (**d**).

Climate matching analysis showed a high probability especially for *P*. *motoro* and *P*. *jabuti* to become established in Indonesia when introduced to new localities (Fig. 2a,b). Individuals that were found were observed to fit perfectly with the predicted suitable areas. Populations of *P*. *motoro* and *P*. *jabuti* can be expected as potentially well-established in Java, Bali, and Nusa Tenggara, and some in Sumatra, Kalimantan, Sulawesi, Maluku Islands and Papua.

### Records in the wild

**Figure 3:**
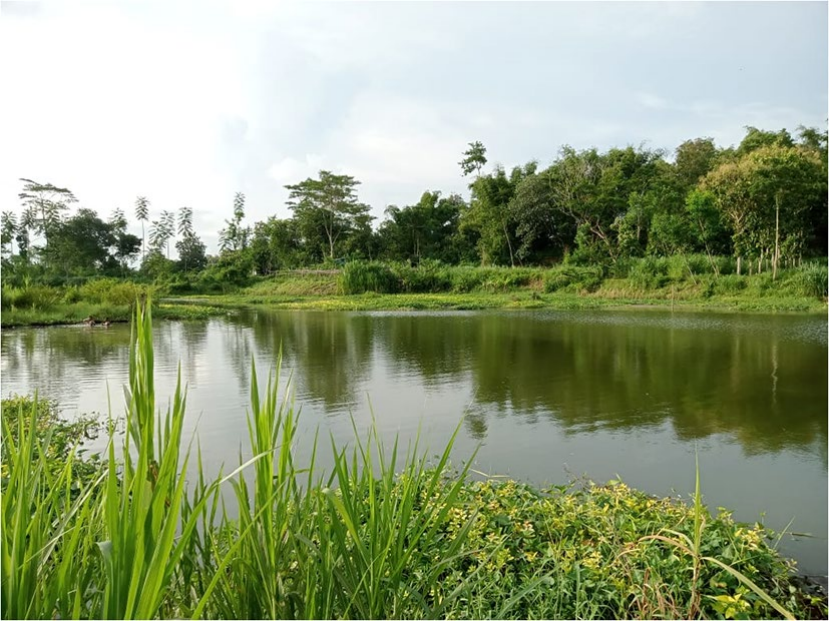
The Brantas River, the locality of a potentially established population of non-native *Potamotrygon* spp. in Java, Indonesia.

In total, 13 individuals of *Potamotrygon* spp. (three morphologically identified as *P*. *motoro*, six as *P*. *jabuti*) were captured by local fishermen using ‘Ayap’ (traditional fishing gear which is similar to big bottom barless dipnet but made from bamboo; the mesh size ranged from 2 to 8 cm; the fisherman use the Ayap from riverbank when the water level is high or during flood, holding it in the water and waiting for fish drifting into the Ayap; when fish where stuck in the net, fisherman lift the Ayap and the fish are caught), angling, castnet, and gillnet in the Brantas River, East Java Province (Fig. 2c, 3, Table 1). Also, newborns and a gravid female of *P*. *jabuti* were recorded and thus, reproduction is occurring. The furthest distance between findings was more than 40 km. The voucher specimen was identified as *Potamotrygon motoro*.

**Table 1:** Records of *Potamotrygon* spp. in Java, Indonesia: species (all of them traded as *P*. *motoro*), quantity (individuals(s)); date (D/M/Y); GPS coordinates; sex (male/female/unknown); size (neonate/subadult/adult); personal inspection (yes/no).

Species	Individual(s)	Date	GPS	Sex	Size	Personal inspection
*Potamotrygon* sp.	1	07 Nov 2021	− 8.153374, 112.204180	Male	Subadult	Yes
*Potamotrygon* sp.	1	15 Nov 2021	− 8.153284, 112.217016	Female	Adult	Yes
*Potamotrygon* sp.	1	22 Nov 2021	− 8.110967, 112.095229	Male	Subadult	Yes
*Potamotrygon motoro*	1*	28 Nov 2021	− 8.1177073, 112.0732019	Male	Subadult	Yes
*Potamotrygon* sp.	1	29 Nov 2021	− 8.145205, 112.252589	Female	Subadult	Yes
*Potamotrygon motoro*	1	30 Nov 2021	− 8.1522527, 112.2709947	Female	Adult	Yes
*Potamotrygon* sp.	1	2 Dec 2021	Uncertain	Female	Subadult	No
*Potamotrygon jabuti*	1	8 Dec 2021	− 8.0890841, 111.9960106	Female	Adult	Yes
*Potamotrygon jabuti*	4	8 Dec 2021	− 8.0890841, 111.9960106	Unknown	Neonate	Yes
*Potamotrygon jabuti*	1	31 Dec 2021	− 8.1176183, 112.0728518	Female	Subadult	Yes
*Potamotrygon motoro*	1	17 March 2022	− 8.1176183, 112.0728518	Female	Subadult	Yes

The voucher specimen is indicated by an asterisk.

**Figure 4:**
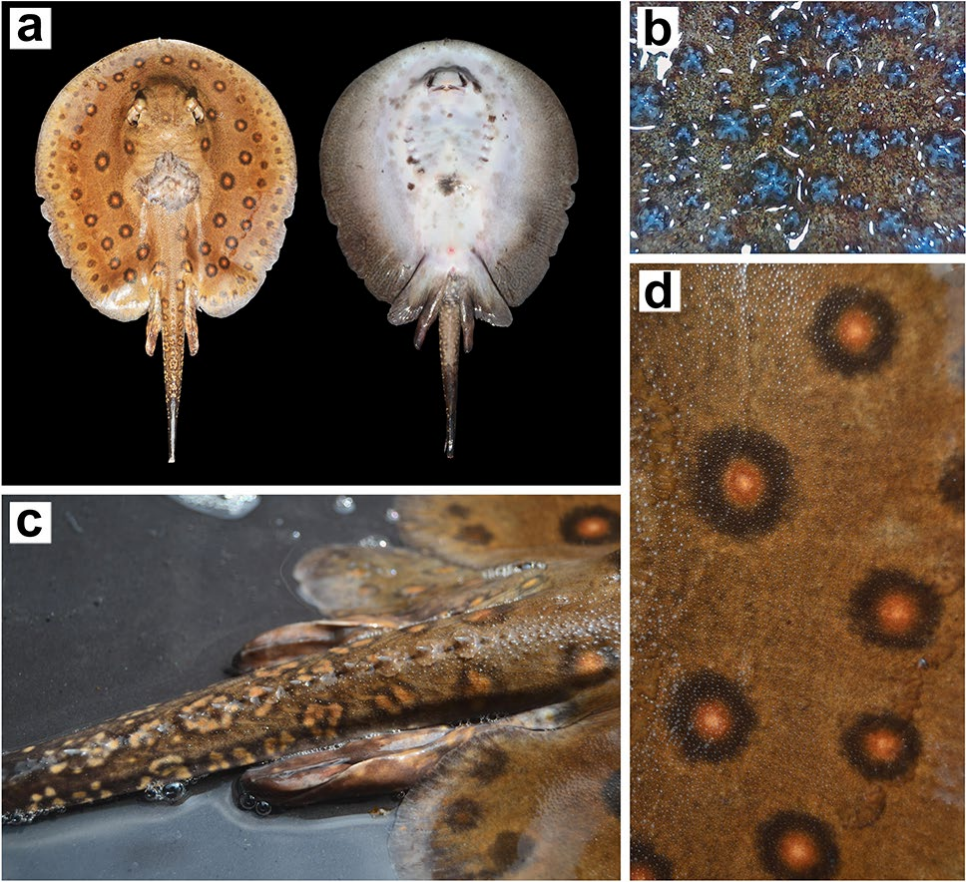
(**a**) *Potamotrygon motoro* captured in Java, Indonesia: Voucher specimen TL 370 mm, DW 230 mm, left (dorsal view) and right (ventral view); (**b**) irregular shape of dermal denticles on voucher specimen (60 ×); (**c**) tricolour ocelli on voucher specimen; (**d**) curved tail spines on voucher specimen.

The voucher specimen (*P*. *motoro*, adult male, total body length 370 mm, disc width 230 mm) was caught on 28 November 2021 using Ayap and kept in an aquarium by a local angler (Fig. 4a). In concordance with previously published morphological analysis^44^, disc was sub-circular with small tips on the anterior margin. Eyes bulging dorsally and relatively large (4% DW). Dorsally covered with tricolour ocelli (Fig. 4d), evenly distributed. The central region of the dorsal disc presents large irregular shapes due to unidentified derm disease (Fig. 4a,b). Pelvic fins are present over the posterior margin of the disc. The tail is relatively short, thick, and punctured on the posterior margin of the sting. One row of curved tail spines with a large base (Fig. 4c). Dermal denticles are present on the dorsal region of the disc and tail, various sizes covered on the disc, but larger denticles present on the central region of the disc, star-shaped denticles with dichotomous ridges present in large denticles, small denticles often with monochotomous ridges or ‘x’ mark-shaped denticles. The ventral disc is whitish with a black and grey irregular shape across the central region, outer margin covered with dark-grey colouration. This voucher specimen is deposited in Museum Zoologicum Bogoriense (MZB), Java, Indonesia, under the coll. No. MZB.26608.

Type habitats of *P*. *motoro* and *P*. *jabuti* in the Brantas River system consist of three areas as the main stream (Brantas River), floodplain, and irrigation canals connected to Brantas River. Water discharges of the main river correlated with dam activity in the upper stream. The connectivity of the floodplain and the main river will be greatly increasing if the water level of the main river was increased. The water discharge in irrigation canals fluctuated following the water level on the reservoir at Njegu Dam and Serut Dam.

We have conducted a rapid qualitative assessment of the habitats in the Brantas River. The Brantas River considers resembling native habitats based on qualitative descriptions of climate (tropic), substrates, and type habitats. The presence of a floodplain and connectivity between the main river and floodplain provides diverse habitat ranges. Muddy and sandy substrates provide similar niches for foraging and burying activities^45^. The habitats in the main river consisted of lacustrine (reservoir) and riverine region (river). Individuals in the lacustrine region (upper stream of Njegu Dam and Serut Dam) were found in areas with mud substrate sporadically covered with leaf litter. Records in the riverine region were found in boulder, pebble, sand, and mud substrates. The voucher specimen was collected on the shore of the riverine region with sand and mud substrates (depth of approximately 0.5–1.5 m). The depth of the main river varies and the observed water clarity range between 0 and 15 cm (wet season). In several sections of the main river, there were observed submerged deposits of sand and pebbles.

The floodplain habitats are located near the main river in the Brantas River system. The gravid female of *P*. *jabuti* with four neonate individuals and one adult male were caught in floodplain habitats. Water depth tends to be shallower (0.5–1.0 m) than the main river and mud dominate the type of substrates. Submerged aquatic plants dominate the edges of the floodplain.

Irrigation canals are connected to the reservoir in the upper stream of the Brantas River. Water clarity tends to fluctuate following reservoir activity (water level, drifted substrates, flood, and dam activity). The majority of substrates are covered by mud and some others are covered by plants and rock. The canal edges were engineered with concrete and rock. There were no confirmed records of *Potamotrygon* spp. in this habitat. On the other hand, local fishermen stated that ‘plenty of small stingrays’ are often seen in the irrigation canals (Lodagung canals). Align with a precautionary approach, we considered this type of habitat as potentially invaded.

Brantas River basin is a national strategic river basin playing a vital role in driving Indonesia’s economy. The Brantas River is the second-longest river (320 km) on the island of Java and crosses the province of East Java. The upper stream of the Brantas River originates from several mountains and springs around the highlands in East Java. The Brantas River flows through various plantation lands, and paddy fields, and is one of the sources of raw material for drinking water in most areas in East Java. Connectivity between main rivers and irrigation canals is located upstream, middle, and downstream. *Potamotrygon* spp. were recorded in the middle (Blitar Regency and Tulungagung Regency) of the Brantas River.

The diets of non-native *Potamotrygon* spp. in the Brantas River are only known from an interview from local anglers. Several anglers use worm bait (*Lumbricus* spp.) and shrimps (*Macrobrachium* spp.) for fishing the *Potamotrygon* spp. The voucher individual was kept in indoor aquarium for several days and had been fed by shrimps and fish (meat-pieces of *Barbonymus* spp. and *Oreochromis* spp.).

Anthropogenic activities in the surveyed area were observed to be diverse and intense. The activities of fishermen, recreational fishing and sand mining were observed in the central part of the Brantas River. These activities are associated with the direct use of water bodies and overlap with the localities of *Potamotrygon* spp. records. Sand mining activities have been carried out in the main river with shallow sand substrates, where *Potamotrygon* spp. were commonly found (for example, locality from confirmed individuals on 22 November 2021 and 31 December 2021 are approximately 50 m from the sand mining site). During the survey of this locality, one fisherman who was stung by a captured *P*. *jabuti* was found. The fisherman was hospitalized for two days after being stung two times (in the left and right forearms). The fisherman described the pain as ‘worse than being stabbed by a dagger’.

### International trade

Of all *P*. *motoro* traded globally, Indonesia imported 2,717 individuals (5.4%), while 122 of them (2.0%) were exported (based on Importer/Exporter-reported quantity of CITES data). Indonesia does not report any trade in *P*. *motoro* to CITES, hence the need to shift focus from exporter to importer-reported data. Between 2017 and 2021, across the globe, all globally reported 5998 *P*. *motoro* were traded, while exporters reported 50,401 were traded. This discrepancy carries through to all *Potamotrygon* species where importers reported 14,868 were traded (2017–2021) while exporters reported 127,489 individuals in the trade (based on data from Fish Quarantine and Inspection Agency (FQIA), Ministry of Marine Affairs and Fisheries Republic of Indonesia).

**Figure 5:**
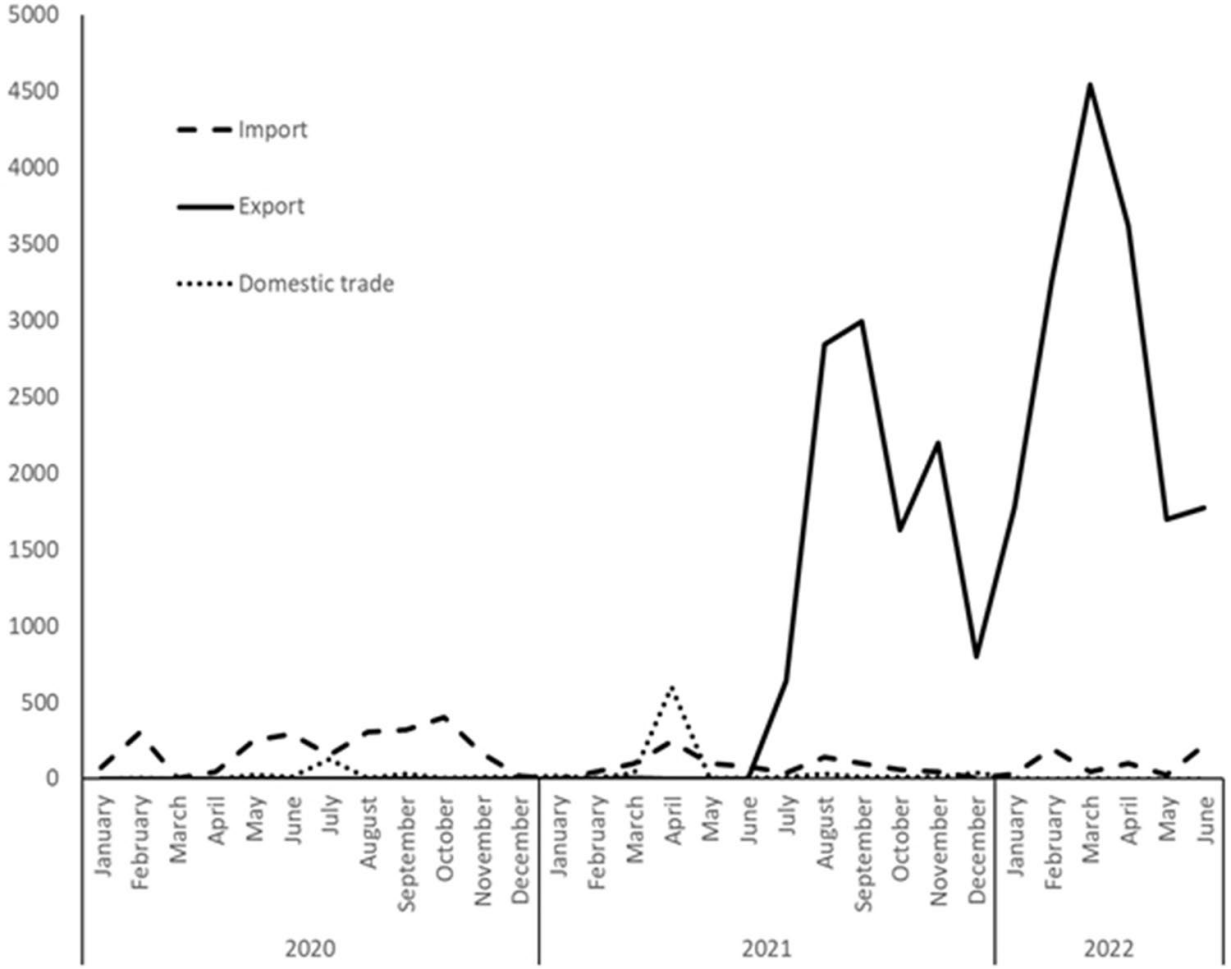
Trade with advertised *Potamotrygon motoro* in Indonesia: import, export, and domestic trade within the period from January 2020 to June 2022.

**Figure 6:**
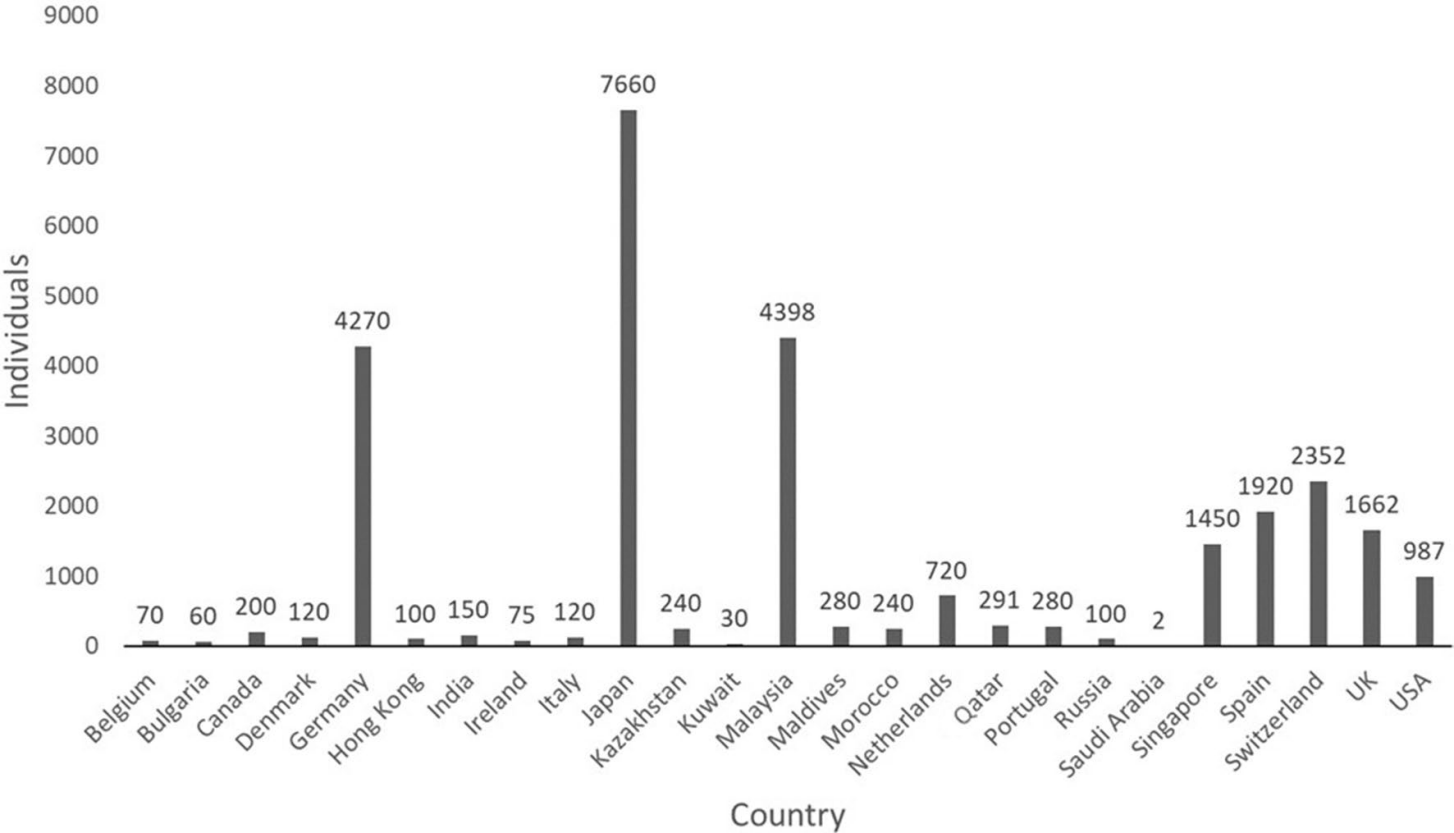
Export of 27,777 individuals traded as *Potamotrygon motoro* from Indonesia within the period from January 2020 to June 2022 with the indicated number of individuals delivered to each customer country.

Data from the Indonesian trade analysis based on data from the Fish Quarantine and Inspection Agency, Ministry of Marine Affairs and Fisheries Republic of Indonesia present a different scenario. In total, 3903 individuals of *P*. *motoro* were imported within the period of January 2020 to June 2022 from Thailand, Taiwan, China, Malaysia, India, and Colombia (the majority of 3525 individuals were from Thailand) and 27,777 individuals were exported to 25 countries (with 7660 individuals delivered to Japan; Figs. 5, 6). Furthermore, 1001 individuals were traded domestically. The first *P*. *motoro* were imported to Indonesia via trade routes from South America by fish entrepreneurs in the 1980s. The first exported individual was delivered to Qatar in March 2021, the largest export was recorded in March 2022 when 4,545 individuals were shipped to 12 countries (Table 2).

**Table 2:** Indonesian trade in *Potamotrygon motoro* (probably more species are traded under the same name) within the period from January 2020 to June 2022 (based on the statistical evidence of Fish Quarantine and Inspection Agency, Ministry of Marine Affairs and Fisheries Republic of Indonesia).

Year	Month	Import	Export	Domestic trade
2020	January	74	0	0
	February	298	0	2
	March	1	0	0
	April	47	0	0
	May	251	0	25
	June	294	0	10
	July	152	0	124
	August	304	0	1
	September	316	0	29
	October	402	0	3
	November	165	0	8
	December	16	0	13
2021	January	0	0	16
	February	47	0	3
	March	101	1	41
	April	246	0	602
	May	101	0	3
	June	81	0	5
	July	35	640	9
	August	143	2845	31
	September	102	2995	8
	October	61	1627	12
	November	47	2195	9
	December	1	800	40
2022	January	30	1780	3
	February	199	3262	0
	March	42	4545	4
	April	102	3617	0
	May	27	1695	0
	June	218	1775	0
Sum		3903	27,777	1001

## Discussion

In Indonesia, an established, self-sustaining population of non-native *Potamotrygon* spp. was found in the Brantas River, Java. This same river also recorded a South American giant arapaima^21^. Obviously, this is what is called a Biodiversity Conservation Paradox^46^ because both mentioned taxa are endangered in their native ranges, but are becoming invasive.

Indonesia has broad ranges of freshwaters from peat water to blackwater (similar to Negro River in low acidity and a huge river system^47^) in Sumatra and Kalimantan (Indonesian part of Borneo) to white-transparent water in three zoogeography areas^48^. The rivers in Borneo and Sumatra are also habitats of native freshwater stingrays (*Fluvitrygon* spp. and *Urogymnus polylepis*)^49,50^. The occurrences of freshwater stingrays (despite different genera and families, potentially similar in their physiology) indicate the potential suitability of natural habitats for to be invaded in the future.

Besides escapes from home aquaria and farms, “Fang Sheng” rite, and pest control^17,21^, a new pathway of non-native species introduction is worth mentioning: affluent people intentionally release expensive animals to demonstrate their wealth and power. There is no clear evidence about the *Potamotrygon* spp. introduction pathway in the Brantas River. Humans value non-natives for a wide range of reasons-aesthetic, culinary, or diverse cultural reasons, among them, financial/social status^51^. With globalization, the trend is that the number of rich Indonesians will increase in the future and thus, maintain the cultural tradition of releasing expensive non-native animals.

Even if climate matching analysis is a preliminary estimation, it showed that the vast majority of Indonesian territory is suitable for further establishment of *P*. *motoro*, *P*. *jabuti*, and other potamotrygonin species. Since aquatic ornamental creatures are known to be transported across the Indonesian territory in huge quantities^40^, it is not surprising that *P*. *motoro* and *P*. *jabuti* are becoming established. The Brantas River resembles native stingray habitats based on qualitative descriptions of climate (tropic), substrates, habitat types, and food resources. Muddy and sandy substrates provide similar niches for foraging and burying activities^45^. The presence of a floodplain and connectivity between the main river and floodplain provides diverse habitat ranges. The record of gravid female at the floodplain confirmed that *Potamotrygon* spp. can use this habitat as nursery ground in the Brantas River. The distribution of *Potamotrygon* spp. was recorded across the floodplain and main river. This stingray is able to overcome artificial barriers (found in three locations separated by artificial barriers such as Upperstream Njegu Dam, Downstream Njegu Dam-Upperstream Serut Dam, and Downstream Serut Dam). It is likely that stingrays crossed the barriers during flood events. A similar situation occurred with introduced *P*. *motoro* and *P*. *falkneri* in Jupiá Dam, Upper Paraná River basin, Southeastern Brazil, attesting to the ability of stingrays to overcome artificial barriers such as hydro power plants^52^.

*Potamotrygon* spp. are a nocturnal predatory fish^53^. The interview-based evidence of broad range of food preferences of recorded non-native *Potamotrygon* spp. have slight similarity with the stomach content of *P*. *motoro* in Negro River^54^. It is known that younger individuals inhabit mostly sandy bottoms of depth no more than four meters while bigger ones migrate between deeper waters during the day and shallow areas at night^45^ but we have no exact data confirming or denying this behaviour also in Indonesia. Because the pigmented and well-camouflaged wild phenotype only is traded in Indonesia and because of burrowing activity, detection of *Potamotrygon* spp. by fishermen and collectors could be difficult. Simply, this species, for having a cryptic colouring, can be easily neglected by locals at least until its abundance will be high. On the other hand, once detected, it can be targeted according to its high price.

There are no native species in Indonesia which can be able to mate with this non-native stingray. On the other hand, negative impacts on native biota and also the local community can be expected. They are regarded by native people in the Amazonia as venomous fishes^55^ and responsible for frequent stings and the envenomation has also occasionally been reported by aquarium traders and owners^56–58^. Especially venomous species can threaten naïve animals and can easily hurt fishermen and all laypersons who will handle these stingrays which is supported by the record of envenomation. For instance, stingray accidents are considered a public health problem in Brazil ^59^. Most of the injuries caused by fish in the country involved freshwater stingrays, especially from the Potamotrygonidae family, and the most affected people are fishermen, who are handling these animals daily, and bathers (children and adults), especially during the dry season in Amazonia, Paraguay and Lower Paraná river basins (i.e., native range)^60,61^. Moreover, accident data in these regions is underestimated because most fishermen do not report or even go to the hospital; some fishermen get used to be frequently sting. In the non-native range (i.e., Upper Paraná River basin), stingray envenomation appeared over the past 20 years. An important natural barrier—the Seven Falls of Guaíra (Paraná State, Southern Brazil)—served as a natural barrier preventing species of the Lower Paraná River to colonize regions of the Upper Paraná River. However, with the creation of the Itaipu hydroelectric dam in 1982, these falls were submerged, allowing the movement of several fish species upstream, including freshwater stingrays that are taking advantage of locks installed at the dam to expand their distribution area^62^. In the Upper course of the Paraná River, injuries are reported by inhabitants and also tourists in municipalities of Mato Grosso do Sul, Paraná and São Paulo states, who are often unaware of the presence of these non-native animals in the area^63^. As is our knowledge, this is the first case of envenomation caused by *Potamotrygon* spp. in the wild outside of their native and non-native locations in South America. Such a situation is alarming because *Potamotrygon* spp. can reach populated areas, where most inhabitants are unaware of how to prevent accidents and to treat wounds they may cause. If we consider the existence of numerous properties by hotels and the intense practice of fishing/tourism activity in the region of Brantas River and throughout the country, it is expected that the negative interaction between humans and *Potamotrygon* spp. will be more intense, causing important changes in epidemiological profiles of envenomation in Indonesia. Further potential encounters between *Potamotrygon* spp. and humans are highly probable and imminent also due to sand mining and fishing in the reported localities in the Brantas River.

Moreover, harmful consequences can be expected due to the predatory feeding behaviour of *Potamotrygon* spp. Diet analysis showed that aquatic invertebrates such as molluscs (both gastropods and bivalves), crustaceans (mainly shrimps from family Palaemonidae), insects (mainly Ephemeroptera and Diptera) and vertebrates such as fish are consumed^64,65^. Also, the foraging behaviour of *Potamotrygon* spp*.* can impact the native fauna. Several species of rays forage disturb the substrate, also known as bioturbation^66,67^, with the use of a tactic we termed “undulate the disc and stir substrate” to uncover insect larvae, crabs, snails and small fish^68^. This activity stirs the substrate particles and discrete sediment clouds are formed near the foraging ray^69^. These clouds can catch the attention of nearby rare *Akysis variegatus*, endangered *Rasbora lateristriata*, protected *Notopterus notopterus* and tadpoles of the vulnerables *Microhyla orientalis* and *Gonocephalus kuhlii* that approach the ray to feed and thus, it can prey on them^70,71^. Freshwater stingrays can also change the diet preferences according to the prey availability in different hydrological seasons. Indeed, this easy adjustment for the available resources can be the main advantage in competition for food with native aquatic organisms. Furthermore, competition for utilizing food and space with native benthic organisms (such as *Hemibagrus* sp. and *Mastacembelus* sp.) is predicted if the populations of *Potamotrygon* spp. explode. Thus, it is obvious that this invader can negatively affect rich Indonesian aquatic biota in general and native, rare, protected, endemic, endangered, and vulnerable species in particular. In line with no predators of potamotrygonin species in their native range^66^, it is also a main problem for these invasive organisms in Indonesia because the control of their populations by native predators is really difficult.

Several parasites such as Digenea, Nematoda and Cestoda are known in *Potamotrygon* spp.^72,73^. Also, branchiuran fish lice *Argulus juparanaensis* was found to infest *P*. *motoro* in its native range^74–76^. Therefore, the parasitic infection of native/endemic species cannot be excluded in the case of *Potamotrygon* spp. spread in Indonesia.

The popularity of various species of *Potamotrygon* as an ornamental fish in Indonesia is obvious because it is produced in significant quantities. In the early part of this species’ history in Indonesia, some individuals were imported from Malaysia and India. The current list of customer countries is wide with Japan, Malaysia and Germany as leaders. No individuals were delivered to the Czech Republic even if this country is perceived as one of the leading producers and traders of ornamental fish globally^37,77,78^. This is probably caused by the significant production of *Potamotrygon* spp. by domestic breeders in the Czech Republic (Dařbujan, H., 2022 pers. comm.). It is obvious that the formal evidence of traded *P*. *motoro* in Indonesia is undervalued by CITES and the real volume of the trade is much higher. This discrepancy in trade evidence increases the potential for *P. motoro* and similar species to be invasive in Indonesia.

What is alarming, the current Indonesian legislative framework is ineffective in preventing the future introductions of this freshwater stingray to new localities and islands across the Indonesian territory^14^. Thus, without significant improvement in the management and regulations, the spread of *Potamotrygon* spp. in Indonesia can be expected to be unlimited for ongoing years. As Reynolds et al.^22^ mentioned, detailed surveys of the trade with potamotrygonids are required for future proposals for CITES listing and we believe that the presented findings can be helpful in this regard.

Presented records align with the habitat use of native potamotrygonids at the Paraná River Basin^45^. Generally, potamotrygonids can adapt to broad ranges of physiochemical parameters^79^ and we assume that this ability is crucial in case of invasion in the Brantas River. Further spread of these species is thus expected and the locality has to be monitored continuously and complemented by physical–chemical parameters measurements. The current condition is predicted as the ‘tip of the iceberg’ invasion event. Thus, continuous monitoring is recommended for this species. Therefore, we emphasize the importance of training health professionals near risk areas, as well as reporting accidents through a kind of “Information System for Notifiable Diseases” as it is carried out in Brazil^61^. According to Brazilian experience, it is recommended to remove fragments, wound cleaning, and immersion of the injured limb in hot water for pain relief (approximately 60 °C—due to the heat-labile properties of some of the toxins in the venom and the vasodilation caused by the hot water helps to counter the intense vasoconstriction and resulting ischemia induced by the venom), antibiotics to prevent bacterial septicemia, gangrene, tetanus, local anaesthetic, and systemic analgesics. Prevention of introductions is the first and most cost-effective management option for the Indonesian aquarium trade. Intensive awareness of importers, wholesalers, retailers, aquarium hobbyists and the general public about why releasing non-native freshwater stingrays is risky for native biota, and why releasing in wild could become a public health problem is crucial. Pamphlets combined with lectures, as well as the dissemination of information through the media, social networks like Facebook, Twitter, Instagram, and WhatsApp and warning signs in places of intense tourism**/**agglomeration of people showing that it is necessary to drag the feet or use a stick or paddle to grope the substrate in order to blows away any freshwater rays are measures that can be effective in preventing the number of accidents by rays in different parts of Indonesia.

Last but not least is to focus on other species of the genus *Potamotrygon* which are offered for sale as ornamental creatures in Indonesia and evaluate their invasion potential and their ability to cause harm to people's health. Additional effort should be placed on the other tropical countries that produce *Potamotrygon* species.

## Methods

Data collection was carried out after the first author received non-formal information from social media regarding the presence of stingrays in the Brantas River, Java (Fig. 2d). An in-depth online desk study was conducted following^21^ in several mass-media and social media outlets (Facebook^®^, www.facebook.com; Instagram^®^, www.instagram.com). A survey including direct observations was carried out in December 2021 by inventorying stingray findings in the Brantas River (Blitar and Tulungagung Regency). Interviews with locals were conducted using the snowball method to qualitatively identify the impact of the invasion on human activities around the Brantas River. In total, 75 people (local non-fishermen who live near the river/locality: such as farmers, ‘tambangan’ people, i.e. person who utilized/organized traditional boat-bridge, local shop keepers; local fishermen: anglers, fishermen) were asked about the occurrence/locality of *Potamotrygon*/freshwater stingray, and only less than 10 providing the specific information about freshwater stingray. Informed consent was obtained from all interviewed people. If possible, individuals observed during the survey were documented and preserved using 96% alcohol. Authors complied with the ARRIVE guidelines. All methods were performed in accordance with the relevant guidelines and regulations. All methods, experiments, protocols, and survey were approved by the ethics committee of Centre for Coastal and Marine Resources Studies at the Bogor Agricultural University (IPB; No. 17/IT3/PL/2012).

Trade information was based on an online survey obtained from two e-commerce platforms (Tokopedia^®^, www.tokopedia.com; Shopee^®^, www.shopee.co.id). Keywords in Bahasa Indonesia and English (pari, pari air tawar, motoro, pari motoro, pari hias, pari black diamond, stingray, stingray motoro, pari marble) were generated and applied to search engines in each platform. The result from each keyword was screened based on the quality of photographs, stock availability, reliability and track record of the stores. The filtered result was synthesized into data and qualitatively described.

The MaxEnt model (v. 3.4.4, https://biodiversityinformatics.amnh.org/open_source/maxent/), a maximum entropy model that is ideally suited to mapping species distributions is commonly used to predict alien species dispersion^80^ since it represents a continuous probability surface of habitat suitability in the target region^19,21,81,82^. This model consists of bioclimatic variables that are characteristics derived from monthly temperatures and rainfall data that reflect yearly patterns, seasonality, and extremes that are crucial for species survival. Bioclimatic factors from the WorldClim database were used to simulate species distribution (v.2.0; https://www.worldclim.org)^83^ with a spatial resolution of 30 s (∼ 1 km^2^). The distribution pattern of aquatic species and environmental factors, particularly temperature, have been discovered to be related^9,84^. These environmental layers were assembled in QGIS 3.24 ‘Tisler’ and released on 13 May 2022 (https://qgis.org/en/site/) to ASCII format for use with the MaxEnt algorithm^85^. We calculated nine bioclimatic variables for both *P*. *motoro* and *P*. *jabuti* (Table 3), these represented the average, extreme and variation of temperature and precipitation and were widely used in ecological niche modelling. MaxEnt was trained using all nine bioclimatic variables with default features and regularization multipliers (Default model), which was based on empirical tuning studies^80^. As the cumulative output, a continuous map was generated and visualized in QGIS 3.24 ‘Tisler’. The MaxEnt model output a threshold value for *P*. *motoro* = 12.74 and *P*. *jabuti* = 16.32. If the value of the climate match reached or exceeded this threshold, this was interpreted as no evidence of climatic constraints to the survival of the species and was shown in red on the map. The value for the area under the receiver operator curve (AUC) for *P*. *motoro* was 0.981 and for *P*. *jabuti*, was 0.982 which means there was a 98% probability for both species that a random selection from presence records had a model score greater than a random selection from the absence records^82^.

**Table 3:** Bioclimatic variables used in the variable selection strategy to build a climate similarity model for *Potamotrygon motoro* and *P*. *jabuti* introduced in Indonesia.

Bioclimatic variables	
BIO1	Annual mean temperature
BIO2	Mean diurnal range (Mean of monthly (max temp − min temp))
BIO3	Isothermality (BIO2/BIO7) (× 100)
BIO4	Temperature seasonality (standard deviation × 100)
BIO5	Max temperature of warmest month
BIO6	Min temperature of coldest month
BIO7	Temperature annual range (BIO5–BIO6)
BIO9	Mean temperature of driest quarter
BIO10	Mean temperature of warmest quarter
BIO11	Mean temperature of coldest quarter

The trade analysis includes a detailed survey of evidence of the domestic market in Indonesia and main customer regions such as Europe with the Czech Republic as a hub^37,77^. Data from the Czech Environmental Inspectorate, (www.cizp.cz), CITES (https://trade.cites.org/) and Fish Quarantine and Inspection Agency (FQIA), Ministry of Marine Affairs and Fisheries Republic of Indonesia were obtained for this purpose and further interpreted.

The species identification of captured individuals was based on morphological analysis and comparison with the latest morphological study^32,86^. Morphological characteristics were measured using a calliper (accuracy 0.1 mm) and microscope images (Dino-lite AM2111). All captured individuals were exploited by the local community as ornamentals or for human consumption with the exception of a voucher specimen.

## Data Availability

The datasets used and analysed during the current study available from the corresponding author on reasonable request.

## References

[1] Simberloff D (2013). Impacts of biological invasions: What’s what and the way forward. Trends. Ecol. Evol..

[2] Pelicice FM (2017). Neotropical freshwater fishes imperilled by unsustainable policies. Fish Fish..

[3] Hulme PE (2021). Unwelcome exchange: International trade as a direct and indirect driver of biological invasions worldwide. One Earth.

[4] Vilizzi L (2021). A global-scale screening of non-native aquatic organisms to identify potentially invasive species under current and future climate conditions. Sci. Total. Environ..

[5] Novák J, Kalous L, Patoka J (2020). Modern ornamental aquaculture in Europe: Early history of freshwater fish imports. Rev. Aquacult..

[6] Akmal SG (2020). Marine ornamental trade in Indonesia. Aquat. Living Resour..

[7] Tapkir SD (2021). Far from home: Tracking the global ornamental fish trade in endangered zebra loach, *Botia striata*, from freshwater ecoregion and biodiversity hotspot in India. J. Nat. Conserv..

[8] Lockwood JL (2019). When pets become pests: The role of the exotic pet trade in producing invasive vertebrate animals. Front. Ecol. Environ..

[9] Magalhães, A. L. B. *et al.* Biotic differentiation in headwater creeks after the massive introduction of non-native freshwater aquarium fish in the Paraíba do Sul River basin, Brazil. *Neotrop. Ichtyol.* **19** (2021).

[10] Patoka J, Bláha M, Kalous L, Kouba A (2017). Irresponsible vendors: non-native, invasive and threatened animals offered for stocking garden ponds. Aquat. Conserv..

[11] Putra MD (2018). *Procambarus clarkii* (Girard, 1852) and crayfish plague as new threats for biodiversity in Indonesia. Aquat. Conserv..

[12] Akmal SG (2022). Culture, trade and establishment of *Polypterus senegalus* in Indonesia with first record of wild populations. Aquacult. Env. Interact..

[13] Bueno ML (2021). Alien fish fauna of southeastern Brazil: Species status, introduction pathways, distribution and impacts. Biol. Invasions.

[14] Patoka J (2018). Invasive aquatic pets: Failed policies increase risks of harmful invasions. Biodivers. Conserv..

[15] Magalhães A (2015). Presence of prohibited fishes in the Brazilian aquarium trade: Effectiveness of laws, management options and future prospects. J. Appl. Ichthyol..

[16] Maceda-Veiga A, Escribano-Alacid J, Martínez-Silvestre A, Verdaguer I, Mac Nally R (2019). What’s next? The release of exotic pets continues virtually unabated 7 years after enforcement of new legislation for managing invasive species. Biol. Invasions.

[17] Patoka J (2020). Two species of illegal South American sailfin catfish of the genus *Pterygoplichthys* well-established in Indonesia. Knowl. Manag. Aquat. Ecosyst..

[18] Herder F (2012). Alien invasion in Wallace’s Dreamponds: Records of the hybridogenic “flowerhorn” cichlid in Lake Matano, with an annotated checklist of fish species introduced to the Malili Lakes system in Sulawesi. Aquat. Invasions.

[19] Akmal SG (2022). Culture, trade and establishment of *Polypterus senegalus* in Indonesia with first record of wild population. Aquacult. Env. Interact..

[20] Magalhães ALB, Vitule JRS (2013). Aquarium industry threatens biodiversity. Science.

[21] Marková J (2020). Conservation paradox of giant arapaima *Arapaima gigas* (Schinz, 1822) (Pisces: Arapaimidae): Endangered in its native range in Brazil and invasive in Indonesia. Knowl. Manag. Aquat. Ecosyst..

[22] Reynolds, J., Hornbrook, E., Stettner, G. & Terrell, R. in *The Elasmobranch Husbandry Manual II: Recent Advances in the Care of Sharks, Rays and their Relatives* Vol. 99 (eds Willson, K. *et al.*) Ch. 11, 99–112 (Ohio Biological Survey, 2017).

[23] Carvalho MD, Loboda TS, Silva J (2016). A new subfamily, Styracurinae, and new genus, *Styracura*, for *Himantura schmardae* (Werner, 1904) and *Himantura pacifica* (Beebe & Tee-Van, 1941) (Chondrichthyes: Myliobatiformes). Zootaxa.

[24] Lucifora LO, Scarabotti PA, Barbini SA (2022). Predicting and contextualizing sensitivity to overfishing in Neotropical freshwater stingrays (Chondrichthyes: Potamotrygonidae). Rev. Fish. Biol. Fisher..

[25] Ng HH, Tan HH, Yeo DC, Ng PK (2010). Stingers in a strange land: South American freshwater stingrays (Potamotrygonidae) in Singapore. Biol. Invasions.

[26] Magalhães, A. L. *et al.* Small size today, aquarium dumping tomorrow: sales of juvenile non-native large fish as an important threat in Brazil. *Neotrop. Ichtyol.* **15** (2017).

[27] Araújo M, Charvet-Almeida P, Almeida MP, Pereira H (2004). Freshwater stingrays (Potamotrygonidae): Status, conservation and management challenges. Cites Org. Doc. AC20.

[28] De Carvalho MR, Lovejoy NR (2011). Morphology and phylogenetic relationships of a remarkable new genus and two new species of Neotropical freshwater stingrays from the Amazon basin (Chondrichthyes: Potamotrygonidae). Zootaxa.

[29] Fontenelle JP, Lovejoy NR, Kolmann MA, Marques FP (2021). Molecular phylogeny for the Neotropical freshwater stingrays (Myliobatiformes: Potamotrygoninae) reveals limitations of traditional taxonomy. Biol. J. Linn. Soc..

[30] Thorson TB, Langhammer JK, Oetinger MI (1983). Reproduction and development of the South American freshwater stingrays, *Potamotrygon circularis* and *P. motoro*. Environ. Biol. Fish..

[31] Charvet-Almeida P, Araújo MLG, Almeida MP (2005). Reproductive aspects of freshwater stingrays (Chondrichthyes: Potamotrygonidae) in the Brazilian Amazon Basin. J. Northwest Atl. Fish..

[32] Loboda TS, Carvalho MR (2013). Systematic revision of the *Potamotrygon motoro* (Müller & Henle, 1841) species complex in the Paraná-Paraguay basin, with description of two new ocellated species (Chondrichthyes: Myliobatiformes: Potamotrygonidae). Neotrop. Ichtyol..

[33] van den Boog, T. *Non-Timber Forest Products: Indigenous Ethnobotanical Knowledge and Livelihood Security in West Suriname*. Master thesis (University of British Columbia, 2017).

[34] de Oliveira AT (2015). Relação entre as populações naturais de arraias de água doce (Myliobatiformes: Potamotrygonidae) e pescadores no baixo rio Juruá, Estado do Amazonas, Brasil. Biota Amazôn..

[35] Fricke, R., Eschmeyer, W. & Van der Laan, R. Eschmeyer’s catalog of fishes: Genera, species, references, http://researcharchive.calacademy.org/research/ichthyology/catalog/fishcatmain.asp (2023).

[36] Camacho-Oliveira RB (2020). DNA barcode reveals the illegal trade of rays commercialized in fishmongers in Brazil. Forensic Sci. Int. Synergy.

[37] Kalous L, Patoka J, Kopecký O (2015). European hub for invaders: Risk assessment of freshwater ornamental fish exported from the Czech Republic. Acta Ichthyol. Piscat..

[38] Patoka J, Kalous L, Kopecký O (2015). Imports of ornamental crayfish: the first decade from the Czech Republic’s perspective. Knowl. Manag. Aquat. Ecosyst..

[39] Akmal SG, Santoso A, Yuliana E, Patoka J (2021). Redclaw crayfish (*Cherax quadricarinatus*): Spatial distribution and dispersal pattern in Java, Indonesia. Knowl. Manag. Aquat. Ecosyst..

[40] Yuliana E (2021). Import, trade and culture of non-native ornamental crayfish in Java. Indonesia. Manag. Biol. Invasions.

[41] Akmal SG, Patoka J (2021). Ornamental aquaculture: Regulation and implementation of digital platforms to support fish trade pathways in Indonesia. Workshop Biodivers. Jevany.

[42] Bomford M, Barry SC, Lawrence E (2010). Predicting establishment success for introduced freshwater fishes: A role for climate matching. Biol. Invasions.

[43] Magalhães ALB, Jacobi CM (2013). Invasion risks posed by ornamental freshwater fish trade to southeastern Brazilian rivers. Neotrop. Ichtyol..

[44] Loboda, T. S. *Revisão Taxonômica e Morfológica de Potamotrygon Motoro (Müller & Henle, 1841) na Bacia Amazônica (Chondrichthyes: Myliobatiformes: Potamotrygonidae)* Master thesis (Universidade de São Paulo, 2010).

[45] Garrone-Neto D, Uieda VS (2012). Activity and habitat use of two species of stingrays (Myliobatiformes: Potamotrygonidae) in the upper Paraná River basin, Southeastern Brazil. Neotrop. Ichtyol..

[46] Vellend M (2017). The biodiversity conservation paradox. Am. Sci..

[47] Duncan WP, Fernandes MN (2010). Physicochemical characterization of the white, black, and clearwater rivers of the Amazon Basin and its implications on the distribution of freshwater stingrays (Chondrichthyes, Potamotrygonidae). Pan-Am. J. Aquat. Sci..

[48] MacKinnon K, Hatta G, Halim H, Mangalik A (1996). The Ecology of Kalimantan.

[49] Iqbal M, Zulkifli H, Yustian I (2018). The valid species and distribution of stingrays (Myliobatiformes: Dasyatidae) in south Sumatera waters, Indonesia. Biovalentia.

[50] Windusari Y, Iqbal M, Hanum L, Zulkifli H, Yustian I (2019). Contemporary distribution records of the giant freshwater stingray *Urogymnus polylepis* in Borneo (Chondrichthyes: Dasyatidae). Ichthyol. Explor. Fres..

[51] Spee LB, Hazel SJ, Dal Grande E, Boardman WS, Chaber A-L (2019). Endangered exotic pets on social media in the Middle East: Presence and impact. Animals.

[52] Júlio Júnior HF, Tós CD, Agostinho ÂA, Pavanelli CS (2009). A massive invasion of fish species after eliminating a natural barrier in the upper rio Paraná basin. Neotrop. Ichtyol..

[53] Garrone-Neto D (2021). Uma área de vida restrita aumenta a vulnerabilidade da raia-de-fogo, *Potamotrygon motoro*, a pesca em um hotspot de biodiversidade da região neotropical. Biota Amazôn..

[54] Shibuya A, Araújo MLGD, Zuanon JAS (2009). Analysis of stomach contents of freshwater stingrays (Elasmobranchii, Potamotrygonidae) from the middle Negro River, Amazonas, Brazil. Pan-Am. J. Aquat. Sci..

[55] Abati PAM (2017). Injuries caused by freshwater stingrays in the Tapajós River Basin: A clinical and sociodemographic study. Rev. Soc. Bras. Med. Trop..

[56] Brisset IB, Schaper A, Pommier P, De Haro L (2006). Envenomation by Amazonian freshwater stingray *Potamotrygon motoro*: 2 cases reported in Europe. Toxicon.

[57] Choa MH (2013). A case report of envenomation and injury by a poisonous spine of a marble motoro (*Potamotrygon motoro*). J. Korean Soc. Clin. Toxicol..

[58] Schiera A, Battifoglio ML, Scarabelli G, Crippa D (2002). Stingray injury in a domestic aquarium. Int. J. Dermatol..

[59] Haddad Junior V, Cardoso JLC, Garrone-Neto D (2013). Injuries by marine and freshwater stingrays: History, clinical aspects of the envenomations and current status of a neglected problem in Brazil. J. Venom. Anim. Toxins Incl. Trop. Dis..

[60] Haddad Junior V, Garrone-Neto D, de Paula Neto JB, de Luna Marques FP, Barbaro KC (2004). Freshwater stingrays: study of epidemiologic, clinic and therapeutic aspects based on 84 envenomings in humans and some enzymatic activities of the venom. Toxicon.

[61] Reckziegel GC, Dourado FS, Garrone Neto D, Haddad V (2015). Lesões causadas por animais aquáticos no Brasil: Uma análise dos dados presentes no sistema de informação para doenças de notificação obrigatória. Rev. Soc. Bras. Med. Trop..

[62] Garrone-Neto, D., Haddad Junior, V., Vilela, M. J. A. & Uieda, V. S. Registro de ocorrência de duas espécies de potamotrigonídeos na região do Alto Rio Paraná e algumas considerações sobre sua biologia. *Biota Neotrop.* **7**, 0–0 (2007).

[63] Garrone-Neto D, Haddad Junior V (2010). Arraias em rios da região Sudeste do Brasil: Locais de ocorrência e impactos sobre a população. Rev. Soc. Bras. Med. Trop..

[64] Silva TB, Uieda VS (2007). Preliminary data on the feeding habits of the freshwater stingrays *Potamotrygon falkneri* and *Potamotrygon motoro* (Potamotrygonidae) from the Upper Paraná River basin, Brazil. Biota Neotrop..

[65] Almeida MPD, Lins PMDO, Charvet-Almeida P, Barthem RB (2010). Diet of the freshwater stingray *Potamotrygon motoro* (Chondrichthyes: Potamotrygonidae) on Marajó Island (Pará, Brazil). Braz. J. Biol..

[66] Shibuya A (2022). A review of the ecological role of the Neotropical freshwater stingrays (Chondrichthyes: Potamotrygoninae). Food Webs.

[67] Shibuya A, Zuanon J, Tanaka S (2012). Feeding behavior of the Neotropical freshwater stingray *Potamotrygon motoro* (Elasmobranchii: Potamotrygonidae). Neotrop. Ichtyol..

[68] Garrone-Neto D, Sazima I (2009). Stirring, charging, and picking: hunting tactics of potamotrygonid rays in the upper Paraná River. Neotrop. Ichtyol..

[69] Garrone-Neto D, Sazima I (2009). The more stirring the better: cichlid fishes associate with foraging potamotrygonid rays. Neotrop. Ichtyol..

[70] Rohman, F. *et al.* Revealing herpetofauna diversity at Brantas River, East Java Indonesia: Evidence of decreasing populations. *Biodiversitas* **23** (2022).

[71] Hasan V (2022). A checklist of native freshwater fish from Brantas River, East Java, Indonesia. Biodiversitas.

[72] Lacerda AC, Takemoto RM, Pavanelli GC (2008). Digenea, Nematoda, Cestoda, and Acanthocephala, parasites in Potamotrygonidae (Chondrichthyes) from the upper Paraná River floodplain, states of Paraná and Mato Grosso do Sul, Brazil. Check List.

[73] Brooks DR, Amato JF (1992). Cestode parasites in *Potamotrygon motoro* (Natterer) (Chondrichthyes: Potamotrygonidae) from southwestern Brazil, including *Rhinebothroides mclennanae* n. sp. (Tetraphyllidea: Phyllobothriidae), and a revised host-parasite checklist for helminths inhabiting neotropical freshwater stingrays. J. Parasitol. Res..

[74] Ivanov VA (2005). A new species of *Acanthobothrium* (Cestoda: Tetraphyllidea: Onchobothriidae) from the ocellate river stingray, *Potamotrygon motoro* (Chondrichthyes: Potamotrygonidae), in Argentina. J. Parasitol..

[75] Souza ACF, Gama CS, da Costa ALP, Costa JF, Viana DC (2020). Índices parasitários de *Brevimulticaecum* sp. (Nematoda: Heterocheilidae) em *Potamotrygon motoro* (Chondrichthyes: Potamotrygonidae) capturados no Arquipélago do Bailique, Macapá. Ens. Ciênc..

[76] Oliveira MSB, Corrêa LL, Oliveira Ferreira D, Neves LR, Tavares-Dias M (2017). Records of new localities and hosts for crustacean parasites in fish from the eastern Amazon in northern Brazil. J. Parasit. Dis..

[77] Novák J (2022). Ornamental aquaculture significantly affected by the “Czech aquarium phenomenon”. Aquaculture.

[78] Evers HG, Pinnegar JK, Taylor MI (2019). Where are they all from?–Sources and sustainability in the ornamental freshwater fish trade. J. Fish Biol..

[79] Duncan WP, Machado RN, Fernandes MN (2021). Environmentally-induced osmoregulation in Neotropical freshwater stingrays (Myliobatiformes: Potamotrygoninae) after controlling for phylogeny. Comp. Biochem. Phys. A.

[80] Phillips SJ, Dudík M (2008). Modeling of species distributions with Maxent: New extensions and a comprehensive evaluation. Ecography.

[81] Yonvitner Y (2020). Enigmatic hotspot of crayfish diversity at risk: Invasive potential of non-indigenous crayfish if introduced to New Guinea. Aquat. Conserv..

[82] Ward DF (2007). Modelling the potential geographic distribution of invasive ant species in New Zealand. Biol. Invasions.

[83] Fick SE, Hijmans RJ (2017). WorldClim 2: New 1-km spatial resolution climate surfaces for global land areas. Int. J. Climatol..

[84] Gallardo B, Errea MP, Aldridge DC (2012). Application of bioclimatic models coupled with network analysis for risk assessment of the killer shrimp, *Dikerogammarus villosus* in Great Britain. Biol. Invasions.

[85] Phillips SJ (2005). A Brief Tutorial on Maxent.

[86] van der Sleen P, Albert JS (2017). Field Guide to the Fishes of the Amazon, Orinoco, and Guianas.

